# The single nucleotide polymorphism rs700518 is an independent risk factor for metabolic syndrome and benign prostatic hyperplasia (MetS‐BPH)

**DOI:** 10.1111/andr.12498

**Published:** 2018-06-05

**Authors:** Z.P. Chen, Y. Yan, C.J. Chen, M. Li, C. Chen, S.C. Zhao, T. Song, T. Liu, C.H. Zou, Q. Xu, X. Li

**Affiliations:** ^1^ Department of Urology Beijing Shijitan Hospital Capital Medical University Beijing China; ^2^ Department of Urology Affiliated Hospital of Zunyi Medical College Zunyi China

**Keywords:** benign prostate hyperplasia, gene polymorphism, metabolism syndrome, sex hormones, single nucleotide polymorphisms

## Abstract

Studies have shown that 48.59% of benign prostate hyperplasia (BPH) is combined with metabolic syndrome (MetS). The mainstream view supports the correlation between MetS and BPH, but the pathogenesis of MetS‐BPH is not fully understood. Four hundred and seventy‐four men, aged 47 years or older, were recruited into this study by consecutive routine physical examination programs, and several parameters were obtained from each participant. Based on the diagnosis of BPH, MetS, and MetS‐BPH, the participants were divided into BPH and Non‐BPH groups, MetS and Non‐MetS groups, as well as MetS‐BPH and Non‐MetS‐BPH groups. The values of the obtained parameters were evaluated using Student's *t*‐test, chi‐square test, and logistic regression analysis. The value of estradiol (E2) was higher in the diseased groups (BPH, MetS, and MetS‐BPH groups) compared with the corresponding control groups (Non‐BPH, Non‐MetS, and Non‐MetS‐BPH groups), and the differences were statistically significant. Also, E2 had an independent association with BPH (OR = 2.286, 95% CI: 1.723–3.593, *p *<* *0.001), MetS (OR = 1.406, 95% CI: 0.585–2.315, *p *<* *0.001), and MetS‐BPH (OR = 1.249, 95% CI: 0.795–1.962, *p *<* *0.001). Regarding SNPs of CYP19A1 gene, both the rs4646 genotypes (CC, CA, and AA) and the rs700518 genotypes (CC, CT, and TT) were present in every group, and all genotypes had statistically significant differences between the diseased and corresponding control groups. However, only the TT genotype of rs700518 was independently associated with BPH, MetS, and MetS‐BPH after adjusting for age. The TT genotype of rs700518 is an independent risk factor for the MetS‐BPH populations, and the CYP19A1 gene regulation of estrogen leads to MetS‐BPH.

## Introduction

Metabolic syndrome (MetS) is a complex medical disorder; it is associated with a constellation of metabolic abnormalities. According to the most widely accepted definition, proposed by the National Cholesterol Education Program's Adult Treatment Panel III (NCEP‐ATPIII), patients with at least three of the following risk factors are considered to have MetS: abdominal obesity (waist circumference >90 cm in men or >85 cm in women), hypertriglyceridemia (triglycerides >150 mg/dL), low high‐density lipoprotein cholesterol (HDL‐C > 40 mg/dL in men and >50 mg/dL in women), high blood pressure (>130/85 mmHg), and a high fasting blood glucose level (>110 mg/dL) (Grundy *et al*., 2005). With the improvement of people's living standard, the incidence of MetS has risen sharply and has become a serious public health problem in modern society (O'Neill & O'Driscoll, 2015). It is estimated that the incidence of MetS in China is more than 25 percent, with more than 100 million patients (Kadihasanoglu & Ozbek, 2015; Ryl *et al*., 2015). Epidemiological studies show that higher socioeconomic status, sedentary lifestyle, central obesity, and high waist circumference are significantly associated with the development of MetS (Dogan *et al*., 2015; Telli *et al*., 2015).

Benign prostatic hyperplasia (BPH) is a common disease of middle‐aged and old men. With the increasing aging of the population, the incidence of BPH is also increasing yearly. BPH severely affects the quality of life of middle‐aged and elderly men (Calais Da Silva *et al*., 1997; Parsons, 2010; Speakman *et al*., 2015; Wang *et al*., 2015). Studies have shown that 48.59% of BPH is combined with MetS (MetS‐BPH), and there is a high degree of correlation between their pathogenesis, occurrence, and development (Pan *et al*., 2014; Gacci *et al*., 2015). The imbalance of sex hormone is an important cause of BPH and the main reason for the emergence of insulin resistance (IR) in MetS. Testosterone replacement therapy (TRT) helps to prevent and improve MetS. It was also found that TRT can improve low urinary tract symptoms (LUTS), make the international prostate symptom score (IPSS) better, decrease residual urine, and increase the maximum urinary flow rate in BPH; however, it does not increase the volume of the prostate and PSA level (Haider *et al*., 2009; Abdollah *et al*., 2011; Corona *et al*., 2014; Cyrus *et al*., 2014). This indicates that a decrease in androgens may be involved in the occurrence of MetS‐BPH.

Aromatase, an enzyme of the cytochrome P450 superfamily, plays a critical role in the biosynthesis of estrogens (C18 steroids) from androgens (C19 steroids) in vertebrate species (Simpson *et al*., 2002). In humans, aromatase is encoded by the CYP19A1 gene, located at chromosome 15q21.2 (Chen *et al*., 1988) and is involved in the oxidation of testosterone to 2β‐, 6β‐, or 15β‐hydroxytestosterone, which are biologically less active than testosterone or dihydroxytestosterone. In fertile women, the ovary represents the major source of circulating estrogens, whereas in men, the testes account for up to 15% of circulating estrogens. The remaining 85% comes from peripheral aromatization of circulating androgen precursors in different tissues, including the adipose tissue, prostate, brain, skin, endothelium, and bone (Gsur *et al*., 2004). Polymorphisms in the CYP19A1 gene showed borderline increased risk of BPH in some studies (Mononen & Schleutker, 2009). Testosterone, the primary circulating androgen in men, can also be metabolized via CYP19/aromatase into the potent estrogen, estradiol‐17β. The prostate is an estrogen target tissue, and estrogens directly and indirectly affect the growth and differentiation of the prostate. Aromatase has important biological functions of maintaining fat metabolism, lipid profile metabolism, glucose metabolism, insulin sensitivity, and sexual behavior (Simpson *et al*., 2002; Santen *et al*., 2009). This indicates that an increase in estrogen may be involved in the occurrence of MetS‐BPH. Also, aromatase, which catalyzes the conversion of androgens into estrogens, may be the common cause of BPH and MetS, and genetic polymorphism may be a common risk factor for BPH, MetS, and sex hormone imbalance (McTernan *et al*., 2000; Berges *et al*., 2011).

The known importance of androgens in the pathogenesis of BPH/BPE, the potential role of CYP19A1 in androgen metabolism, and the association between both polymorphisms and BPH risk prompted us to investigate the association between genetic polymorphisms in the CYP19A1 gene and several clinical and laboratory BPH parameters in a population‐based sample. However, at present, we are not yet fully aware of the pathogenesis of MetS‐BPH. Sex hormone imbalance may be the bridge between MetS and BPH, but whether there is a common pathway remains to be elucidated. Sex hormone‐related gene polymorphism and the correlation between MetS and BPH need further research.

We had investigated whether MetS is related to lower urinary tract symptoms (LUTS) resulting from benign prostate hyperplasia (BPH) (Zhao *et al*., 2016). In this study, after detecting the middle‐aged male crowd SNPs (rs4646 and rs700518) of CYP19A1 gene, multiple sex hormone indexes, as well as BPH‐ and MetS‐related parameters, we compared their distribution, variation, and correlation between BPH and Non‐BPH, MetS and Non‐MetS, as well as MetS‐BPH and Non‐MetS‐BPH groups, to further confirm whether the CYP19A1 gene polymorphisms clinically influence MetS‐BPH by the regulation of some sex hormone (especially estrogen) metabolism.

## Materials and Methods

### Study population

This study is a case–control study. The institutional review board of the Beijing Shijitan Hospital approved this study on September 2014, and all procedures performed in this study involving human participants were in accordance with the ethical standards of the institutional committee and with the 1964 Helsinki declaration and its later amendments or comparable ethical standards. From October 2014 to December 2014, 871, community elderly male (age, 47–88 years) residents who had an international prostate symptom score (IPSS) evaluation and had consecutively participated in prostate examinations at Beijing Shijitan Hospital were recruited into this study. To minimize potential confounding factors and bias, the participants who had a history of prostate or urethral surgery; those who had been diagnosed with urologic diseases, including urethral stricture, urologic infections, malignancy, or neurogenic bladder; and those who had been administrated drugs, including anticholinergics, 5α‐reductase inhibitors, phosphodiesterase‐5 inhibitors, and hormone replacement therapy were excluded from the study. In summary, 86 men suffered one or several of the above conditions; 16 men refused to undergo a transrectal ultrasound examination of the prostate; 11 men did not finish the IPSS questionnaire; 196 patients took BPH‐related medications, and 32 patients were diagnosed with prostate cancer by prostate biopsy. Moreover, in 56 men whose blood samples were extracted, the DNA was not up to standard. Finally, the remaining 474 participants were included in this study. Detailed informed consents were obtained from all subjects before enrollment.

### Blood specimens’ collection, detection, and cryopreservation

All the blood specimens were obtained from the subjects in the morning after an overnight fast. The serum prostate‐specific antigen (PSA) levels were determined using radioimmunoassay. The serum total testosterone (TT), estradiol (E2), dihydrotestosterone (DHT), insulin (INS), and androgen‐binding globulin (SHBG) were determined by enzyme‐linked immunosorbent assay (ELISA) at Beijing Huada Protein Research and Development Center Co., Ltd. Molecular testing laboratory (Beijing Protein Innovation) and the DRG^®^ Elisa kits were used. The biochemical analyses, including fasting plasma glucose (FPG), triglycerides, HDL‐C, and total cholesterol (TC), were determined by fully automatic biochemical analyzer. The insulin resistance index (HOMA‐IR) was calculated using the following formula: [FPG(mmol/L) × INS(mIU/L) ÷ 22.5], and the value of HOMA‐IR >2.69 was defined as the occurrence of insulin resistance. The genomic DNA was extracted from peripheral blood (cryopreserved in advance) using standard phenol/chloroform method.

### SNP selection and genotyping

We selected two SNPs, including rs4646 at 3′UTR (A161C) and rs700518 which is a synonymous SNP (C264T) located in exon 3 (Valine 80) of the CYP19A1 gene (http://www.ncbi.nlm.nih.gov/SNP), for analysis. Peripheral blood samples from each subject were stored in ethylenediaminetetraacetic acid in blood sampling tubes at −20 °C. Genomic DNA was extracted using standard phenol/chloroform method. PCR‐amplified target sequences included rs4646 (5′‐GACCAAGCTAGGTGCTATTG‐3′ and 5′‐GACCAAGCTAGGTGCTATTT‐3′, 141 base pairs) and rs700518 (5′‐ACTCGCATGAATTCTCCATAC‐3′ and 5′‐ACTCGCATGAATTCTCCATAT‐3′, 143 base pairs). The reaction contained 20 ng (2 μL) of genomic DNA. The universal reaction conditions were the 200‐short‐cycle‐program using two cycling loops, one of five cycles that sits inside a loop of 40 cycles. These two loops result in a 200‐cycle program. The sample is denatured at 94 °C. Strands are annealed at 52 °C for 5 sec and extended at 80 °C for 5 sec. The annealing and extension cycle are repeated four more times, making a total of five cycles, and then looped back to a 94 °C denaturing step for 5 sec. The 5‐cycle annealing and extension steps with the single denaturing step are repeated an additional 39 times, making a total of 40 cycles. The 40 cycles of the 5‐cycle annealing and extension steps equate to a total of 200 cycles (5 × 40). A final extension is carried out at 72 °C for 3 min, and then, the sample is cooled to 4 °C. Matrix‐assisted laser resolution ionization flight time mass spectrometry (MALDI‐TOF‐MS) is used for detection. typer 4.0 software (Agena Bioscience, San Diego, CA 92121, USA) was used to detect the mass spectra, and the genotypes of each sample target site were read according to the mass spectra. Assay design, DNA isolation, PCR amplification, direct sequencing, and analysis were performed with the iPLEX MassARRAY platform (Sequenom) (Beaulieu, 2003; Tang *et al*., 2004).

### Assessment and definition of BPH

The subjects’ medical histories were collected using a standardized structured questionnaire. The Chinese version of the IPSS was administered to the subjects to evaluate urinary symptoms. The total prostate volume (TPV) was measured using transrectal ultrasonography, and TPV was calculated using the prolate ellipse formula (transverse × anteroposterior × cephalocaudal diameter × π/6). The maximum urinary flow rate (Qmax) was determined by uroflowmetry at a voided volume of >150 mL. All subjects underwent digital examinations of the rectum to exclude palpable prostatic nodules. According to the results from the placebo‐arm study of the Medical Therapy of Prostatic Symptoms study (MTOPS), TPV >20 cm^3^ was defined as BPH, and the defined predictors for clinical BPH progression included a TPV ≥31 cm^3^, Qmax <10.6 mL/sec, PSA ≥1.6 ng/mL, and age of 45 years or older (Allen *et al*., 2001).

### Definition of MetS

MetS was diagnosed using the 2005 National Cholesterol Education Program‐Adult Treatment Panel III (NCEP‐ATP III) criteria for Asian Americans (Grundy *et al*., 2005). The modified NCEP‐ATP III defined MetS as the simultaneous occurrence of at least three of the following five risk factors: (i) waist circumference ≥90 cm, (ii) triglycerides ≥1.70 mmol/L or drug treatment for elevated triglycerides, (iii) HDL‐C < 1.04 mmol/L or drug treatment for reduced HDL‐C, (iv) blood pressure ≥130/85 mmHg or antihypertensive drug treatment with a history of hypertension, and (v) fasting plasma glucose (FPG) ≥6.1 mmol/L, 2‐h post‐prandial blood glucose (2hPG) ≥7.8 mmol/L or drug treatments for elevated glucose.

### Definition of MetS‐BPH and eucrasia

MetS‐BPH refers to the above diagnostic criteria of MetS and BPH, and the occurrence of MetS simultaneously with BPH. Eucrasia refers to the indicators in this study that do not have diagnostic MetS, BPH, and other diseases, and the physical examination indicators are normal.

### Data extraction

The following parameters were obtained from each participant: age (year), TT, E2, DHT, INS, FPG, SHBG, IPSS, Qmax, PSA, TPV, HOMA‐IR, SNPs (rs700518, rs4646) of CYP19A1 gene, whether BPH was diagnosed, whether MetS was diagnosed, whether MetS‐BPH was diagnosed, and whether eucrasia was diagnosed.

### Statistical analyses

Statistical analyses were performed using Statistical Package for the Social Science (SPSS Inc., Chicago, IL, USA) version 18.0 for windows (SPSS Inc.). The selected characteristics were expressed as mean and standard deviation (mean ± SD) as well as percentage (%) for comparison between BPH and Non‐BPH groups, MetS and Non‐MetS groups, as well as MetS‐BPH and Non‐MetS‐BPH groups. Student's *t*‐test was used for continuous variables; allelic and genotypic associations were evaluated by chi‐square test; multivariate‐adjusted odds ratios (ORs) and 95% confidence intervals (CIs) were simultaneously estimated by logistic regression analyses. Differences were considered statistically significant by a *p* value of <0.05.

## Results

### Patients characteristics

The principal characteristics of our study population are listed in Table 1. A total of 474 men (69.26 ± 8.28 years; 47–88 years) were analyzed. The mean ± standard deviation (SD) of the parameters was as follows: TT—4.64 ± 2.08 ng/mL, E2—38.62 ± 17.43 pg/mL, INS—51.75 ± 30.96 μIU/mL, FPG—5.55 ± 1.35 mmol/L, SHBG—70.20 ± 34.90 nmol/L, DHT—381.39 ± 244.68 pg/mL, IPSS—9.30 ± 8.22, Qmax—17.08 ± 5.57 mL/sec, PSA—1.38 ± 1.38 ng/mL, TPV—22.82 ± 6.76 mL, and HOMA‐IR—1.84 ± 1.24. BPH, MetS, MetS‐BPH, and eucrasia accounted for 50.42% (239/474), 7.81% (37/474), 19.83% (94/474), and 21.94% (104/474), respectively. These data suggest a typical population of elderly men generated via population‐based sampling.

**Table 1:** Principal cohort characteristics (*n *=* *474)

Variables	Mean	Standard deviation	Min	Max
Age, years	69.26	8.28	47	88
TT, ng/mL	4.64	2.0	0.22	32.50
E2, pg/mL	38.62	17.43	5.88	161.86
INS, μIU/mL	51.75	30.96	6.59	229.09
FPG, mmol/L	5.55	1.35	3.62	14.52
SHBG, nmol/L	70.20	34.90	11.29	242.80
DHT, pg/mL	381.39	244.68	60.37	2761.41
IPSS	9.30	8.22	0	34
Qmax, mL/sec	17.08	5.57	5.90	36.40
PSA, ng/mL	1.38	1.38	0.03	9.51
TPV, mL	22.82	6.76	7.60	51.20
HOMA‐IR	1.84	1.24	0.21	10.30
BPH (%)	50.42 (239/474)			
MetS (%)	7.81 (37/474)			
MetS‐BPH (%)	19.83 (94/474)			
Eucrasia (%)	21.94 (104/474)			

TT, total testosterone; E2, estradiol; DHT, dihydrotestosterone; INS, insulin; FPG, fasting plasma glucose; SHBG, androgen‐binding globulin; IPSS, international prostate symptom score; Qmax, maximum urinary flow rate; PSA, prostate‐specific antigen; TPV, total prostate volume; HOMA‐IR, insulin resistance index; BPH, benign prostatic hyperplasia; MetS, metabolic syndrome; MetS‐BPH, BPH combined with MetS.

### Association between the disease of BPH and the related parameters of sex hormone, BPH, and MetS

As shown in Table 2, with respect to the comparison between the BPH and Non‐BPH groups, the following parameters had statistically significant differences: age (70.03 ± 8.07 and 67.45 ± 8.51 years, *p *=* *0.002), E2 (38.85 ± 18.92 and 37.09 ± 13.29 pg/mL, *p *=* *0.042), FPG (5.73 ± 1.58 and 5.47 ± 1.23 mmol/L, *p *=* *0.049), IPSS (11.56 ± 8.41 and 3.97 ± 4.43, *p *<* *0.001), TPV (24.03 ± 6.84 and 19.95 ± 5.60 mL, *p *<* *0.001), and HOMA‐IR (2.01 ± 1.43 and 1.76 ± 1.14, *p *=* *0.048). The following parameters had independent associations with BPH: E2 (OR = 2.286, 95% CI: 1.723–3.593, *p *<* *0.001), IPSS (OR = 1.187, 95% CI: 1.138–1.238, *p *<* *0.001), TPV (OR = 1.108, 95% CI: 1.062–1.157, *p *<* *0.001), and Qmax (OR = 1.061, 95% CI: 1.013–1.111, *p *=* *0.012). Also, TT, INS, SHBG, DHT, and PSA did not have statistically significant differences with respect to the comparison between the BPH and Non‐BPH groups (in all cases, *p *>* *0.05).

**Table 2:** Association between the disease of BPH and the related parameters of sex hormone, BPH, and MetS

Parameters	Number	BPH	*p‐*value^c^	OR	*p‐*value^d^	95% CI
Age, years						
(+)^a^	333	70.03 ± 8.07	**0.002**	1.030	0.065	0.998–1.062
(−)^b^	141	67.45 ± 8.51				
TT, ng/mL						
(+)^a^	333	4.54 ± 2.25	0.985	0.979	0.744	0.862–1.112
(−)^b^	141	4.64 ± 1.63				
E2, pg/mL						
(+)^a^	333	38.85 ± 18.92	**0.042**	2.286	**<0.001**	1.723–3.593
(−)^b^	141	37.09 ± 13.29				
INS, μIU/mL						
(+)^a^	333	50.17 ± 27.82	0.088	0.974	0.103	0.943–1.005
(−)^b^	141	55.48 ± 37.19				
FPG, mmol/L						
(+)^a^	333	5.73 ± 1.58	**0.049**	0.726	0.055	0.523–1.007
(−)^b^	141	5.47 ± 1.23				
SHBG, nmol/L						
(+)^a^	333	71.27 ± 34.35	0.318	0.997	0.563	0.989–1.006
(−)^b^	141	67.69 ± 36.18				
DHT, pg/mL						
(+)^a^	333	372.86 ± 252.39	0.224	1.000	0.950	0.999–1.001
(−)^b^	141	401.47 ± 225.07				
IPSS						
(+)^a^	333	11.56 ± 8.41	**<0.001**	1.187	**<0.001**	1.138–1.238
(−)^b^	141	3.97 ± 4.43				
Qmax, mL/sec						
(+)^a^	333	17.06 ± 5.59	0.932	1.061	**0.012**	1.013–1.111
(−)^b^	141	17.11 ± 5.55				
PSA, ng/mL						
(+)^a^	333	1.37 ± 1.35	0.864	0.975	0.766	0.828–1.149
(−)^b^	141	1.39 ± 1.44				
TPV, mL						
(+)^a^	333	24.03 ± 6.84	**<0.001**	1.108	**< 0.001**	1.062–1.157
(−)^b^	141	19.95 ± 5.60				
HOMA‐IR						
(+)^a^	333	2.01 ± 1.43	**0.048**	1.457	0.373	0.637–3.331
(−)^b^	141	1.76 ± 1.14				

BPH, benign prostatic hyperplasia; OR, odds ratio; CI, confidence interval; TT, total testosterone; E2, estradiol; DHT, dihydrotestosterone; INS, insulin; FPG, fasting plasma glucose; SHBG, androgen‐binding globulin; IPSS, international prostate symptom score; Qmax, maximum urinary flow rate; PSA, prostate‐specific antigen; TPV, total prostate volume; HOMA‐IR, insulin resistance index; MetS, metabolic syndrome. The boldface represents statistical significance (*p *<* *0.05).

a On behalf of the BPH.

b On behalf of the Non‐BPH.

c Student's *t*‐test.

d Multivariate logistic regression analysis.

### Association between the disease of MetS and the related parameters of sex hormone, BPH, and MetS

As shown in Table 3, with respect to the comparison between the MetS and Non‐MetS groups, the following parameters had statistically significant differences: age (67.35 ± 8.57 and 69.99 ± 8.06 years, *p *=* *0.003), TT (3.95 ± 1.31 and 4.90 ± 2.26 ng/mL, *p* < 0.001), E2 (40.14 ± 18.29 and 38.04 ± 17.08 pg/mL, *p* = 0.048), INS (60.12 ± 32.01 and 48.56 ± 29.99 μIU/mL, *p *<* *0.001), FPG (6.24 ± 1.73 and 5.28 ± 1.06 mmol/L, *p *<* *0.001), SHBG (57.41 ± 25.15 and 75.09 ± 36.85 nmol/L, *p *<* *0.001), IPSS (11.03 ± 8.38 and 8.64 ± 8.08, *p *=* *0.006), Qmax (15.33 ± 5.39 and 17.74 ± 5.50 mL/sec, *p *<* *0.001), PSA (1.14 ± 0.90 and 1.47 ± 1.51 ng/mL, *p *=* *0.004), and HOMA‐IR (2.38 ± 1.48 and 1.63 ± 1.06, *p *<* *0.001). E2 (OR = 1.406, 95% CI: 0.585–2.315, *p *<* *0.001) was independently associated with MetS. Also, DHT and TPV did not have statistically significant differences with respect to the comparison between the MetS and Non‐MetS groups (All *p *>* *0.05).

**Table 3:** Association between MetS and the related parameters of sex hormone, BPH, and MetS

Parameters	Number	MetS	*p‐*value^c^	OR	*p‐*value^d^	95% CI
Age, years						
(+)^a^	131	67.35 ± 8.57	**0.003**	0.953	0.147	0.892–1.017
(−)^b^	343	69.99 ± 8.06				
TT, ng/mL						
(+)^a^	131	3.95 ± 1.31	**<0.001**	0.729	0.204	0.447–1.188
(−)^b^	343	4.90 ± 2.26				
E2, pg/mL						
(+)^a^	131	40.14 ± 18.29	**0.048**	1.406	**<0.001**	0.585–2.315
(−)^b^	343	38.04 ± 17.08				
INS, μIU/mL						
(+)^a^	131	60.12 ± 32.01	**<0.001**	0.986	0.619	0.931–1.044
(−)^b^	343	48.56 ± 29.99				
FPG, mmol/L						
(+)^a^	131	6.24 ± 1.73	**<0.001**	1.327	0.309	0.770–2.288
(−)^b^	343	5.28 ± 1.06				
SHBG, nmol/L						
(+)^a^	131	57.41 ± 25.15	**<0.001**	0.993	0.521	0.970–1.015
(−)^b^	343	75.09 ± 36.85				
DHT, pg/mL						
(+)^a^	131	391.84 ± 239.19	0.561	0.999	0.373	0.996–1.001
(−)^b^	343	377.38 ± 246.98				
IPSS						
(+)^a^	131	11.03 ± 8.38	**0.006**	1.060	0.232	0.964–1.165
(−)^b^	343	8.64 ± 8.08				
Qmax, mL/sec						
(+)^a^	131	15.33 ± 5.39	**<0.001**	0.966	0.433	0.885–1.054
(−)^b^	343	17.74 ± 5.50				
PSA, ng/mL						
(+)^a^	131	1.14 ± 0.90	**0.004**	1.045	0.803	0.737–1.482
(−)^b^	343	1.47 ± 1.51				
TPV, mL						
(+)^a^	131	23.43 ± 7.17	0.244	1.045	0.285	0.964–1.132
(−)^b^	343	22.58 ± 6.59				
HOMA‐IR						
(+)^a^	131	2.38 ± 1.48	**<0.001**	1.721	0.497	0.359–8.262
(−)^b^	343	1.63 ± 1.06				

BPH, benign prostatic hyperplasia; OR, odds ratio; CI, confidence interval; TT, total testosterone; E2, estradiol; DHT, dihydrotestosterone; INS, insulin; FPG, fasting plasma glucose; SHBG, androgen‐binding globulin; IPSS, international prostate symptom score; Qmax, maximum urinary flow rate; PSA, prostate‐specific antigen; TPV, total prostate volume; HOMA‐IR, insulin resistance index; MetS, metabolic syndrome. The boldface represents statistical significance (*p *<* *0.05).

a On behalf of the MetS.

b On behalf of the Non‐MetS.

c Student's *t*‐test.

d Multivariate logistic regression analysis.

### Association between the disease of MetS‐BPH and the related parameters of sex hormone, BPH, and MetS

As shown in Table 4, the following parameters had statistically significant differences regarding the comparison between the MetS‐BPH and Non‐Mets‐BPH groups: TT (3.96 ± 1.34 and 4.81 ± 2.20 ng/mL, *p *<* *0.001), E2 (42.06 ± 19.22 and 37.77 ± 16.87 pg/mL, *p *=* *0.032), INS (58.38 ± 29.98 and 50.11 ± 31.02 μIU/mL, *p *=* *0.019), FPG (6.07 ± 1.53 and 5.42 ± 1.27 mmol/L, *p *<* *0.001), SHBG (59.95 ± 25.44 and 72.74 ± 36.45 nmol/L, *p *<* *0.001), IPSS (13.50 ± 8.07 and 8.27 ± 7.93, *p *<* *0.001), Qmax (15.03 ± 5.18 and 17.58 ± 5.55 mL/sec, *p *<* *0.001), PSA (1.06 ± 0.62 and 1.45 ± 1.50 ng/mL, *p *<* *0.001), TPV (24.48 ± 7.06 and 22.41 ± 6.63 mL, *p *=* *0.011), and HOMA‐IR (2.27 ± 1.44 and 1.73 ± 1.16, *p *=* *0.002). The following parameters had independent associations with MetS‐BPH: TT (OR = 0.675, 95% CI: 0.525–0.866, *p *=* *0.002), E2 (OR = 1.249, 95% CI: 0.795–1.962, *p *<* *0.001), IPSS (OR = 1.080, 95% CI: 1.045–1.115, *p *<* *0.001), Qmax (OR = 0.945, 95% CI: 0.897–0.995, *p *=* *0.031), PSA (OR = 0.673, 95% CI: 0.485–0.933, *p *=* *0.018), and HOMA‐IR (OR = 1.234, 95% CI: 0.489–3.117, *p *=* *0.036). Also, age and DHT did not have statistically significant differences when the MetS and Non‐MetS groups were compared (All *p *>* *0.05).

**Table 4:** Association between MetS‐BPH and the related parameters of sex hormone, BPH, and MetS

Parameters	Number	MetS‐BPH	*p‐*value^c^	OR	*p‐*value^d^	95% CI
Age, years						
(+)^a^	94	68.55 ± 8.07	0.348	0.977	0.183	0.944–1.011
(−)^b^	380	69.43 ± 8.34				
TT, ng/mL						
(+)^a^	94	3.96 ± 1.34	**<0.001**	0.675	**0.002**	0.525–0.866
(−)^b^	380	4.81 ± 2.20				
E2, pg/mL						
(+)^a^	94	42.06 ± 19.22	**0.032**	1.249	**<0.001**	0.795–1.962
(−)^b^	380	37.77 ± 16.87				
INS, μIU/mL						
(+)^a^	94	58.38 ± 29.98	**0.019**	0.993	0.673	0.959–1.028
(−)^b^	380	50.11 ± 31.02				
FPG, mmol/L						
(+)^a^	94	6.07 ± 1.53	**<0.001**	1.298	0.124	0.931–1.811
(−)^b^	380	5.42 ± 1.27				
SHBG, nmol/L						
(+)^a^	94	59.95 ± 25.44	**<0.001**	1.001	0.828	0.990–1.012
(−)^b^	380	72.74 ± 36.45				
DHT, pg/mL						
(+)^a^	94	393.52 ± 261.83	0.611	1.000	0.426	1.000–1.001
(−)^b^	380	378.38 ± 240.51				
IPSS						
(+)^a^	94	13.50 ± 8.07	**<0.001**	1.080	**< 0.001**	1.045–1.115
(−)^b^	380	8.27 ± 7.93				
Qmax, mL/sec						
(+)^a^	94	15.03 ± 5.18	**<0.001**	0.945	**0.031**	0.897–0.995
(−)^b^	380	17.58 ± 5.55				
PSA, ng/mL						
(+)^a^	94	1.06 ± 0.62	**<0.001**	0.673	**0.018**	0.485–0.933
(−)^b^	380	1.45 ± 1.50				
TPV, mL						
(+)^a^	94	24.48 ± 7.06	**0.011**	1.024	0.216	0.986–1.063
(−)^b^	380	22.41 ± 6.63				
HOMA−IR						
(+)^a^	94	2.27 ± 1.44	**0.002**	1.234	**0.036**	0.489–3.117
(−)^b^	380	1.73 ± 1.16				

BPH, benign prostatic hyperplasia; OR, odds ratio; CI, confidence interval; TT, total testosterone; E2, estradiol; DHT, dihydrotestosterone; INS, insulin; FPG, fasting plasma glucose; SHBG, androgen‐binding globulin; IPSS, international prostate symptom score; Qmax, maximum urinary flow rate; PSA, prostate‐specific antigen; TPV, total prostate volume; HOMA‐IR, insulin resistance index; MetS, metabolic syndrome; MetS‐BPH, BPH combined with MetS. The boldface represents statistical significance (*p *<* *0.05).

a On behalf of the MetS‐BPH.

b On behalf of the Non‐MetS‐BPH.

c Student's *t*‐test.

d Multivariate logistic regression analysis.

### The genotypes and allele distributions of rs700518 and rs4646 in BPH and Non‐BPH groups and the comparison between the two groups

The genotypic distribution of rs4646 and rs700518 in the study subjects was in accordance with Hardy–Weinberg equilibrium (data not shown). As shown in Table 5, regarding the SNPs of CYP19A1 gene, the genotypes CC, CA, and AA of rs4646 were present in the BPH and Non‐BPH groups, accounting for 50.15% (167/333) and 51.77% (73/141); 42.34% (141/333) and 41.13% (58/141); and 7.51% (25/333) and 7.09% (10/141), respectively. The genotypes CC, CT, and TT of rs700518 were present in the BPH and Non‐BPH groups, accounting for 21.02% (70/333) and 19.15% (27/141); 52.25% (174/333) and 49.65% (70/141); and 26.73% (89/333) and 31.20% (44/141), respectively. All genotypes had statistically significant differences between the diseased and control groups (in all cases, *p *≤* *0.014). However, only the genotype TT of rs700518 was independently associated with BPH (OR = 1.324, 95% CI: 0.811–2.163, *p *=* *0.026) after adjusting for age.

**Table 5:** The genotypes and allele distributions of rs700518 and rs4646 in BPH and Non‐BPH groups and the comparison between the two groups

SNP	Type	BPH, ratio (%)	Non‐BPH, ratio (%)	*p*‐value^a^	Model	OR (95% CI)	*p*‐value^b^
rs4646	CC	167/333 (50.15)	73/141 (51.77)	**<0.001**		1.181 (0.745–1.871)	0.479
	CA	141/333 (42.34)	58/141 (41.13)	**<0.001**		.	.
	AA	25/333 (7.51)	10/141 (7.09)	**0.014**	Allele	0.835 (0.362–1.926)	0.673
rs700518	CC	70/333 (21.02)	27/141 (19.15)	**<0.001**	Allele	0.882 (0.500–1.555)	0.665
	CT	174/333 (52.25)	70/141 (49.65)	**<0.001**		.	.
	TT	89/333 (26.73)	44/141 (31.20)	**<0.001**		1.324 (0.811–2.163)	**0.026**

BPH, benign prostatic hyperplasia; SNP, single nucleotide polymorphisms; OR, odds ratio; CI, confidence interval. The boldface represents statistical significance (*p *<* *0.05).

a Chi‐square test.

b Multivariate logistic regression analysis, adjusted for age.

### The genotypes and allele distributions of rs700518 and rs4646 in MetS and Non‐MetS group and the comparison between the two groups

As shown in Table 6, with respect to the SNPs of CYP19A1 gene, the genotypes CC, CA, and AA of rs4646 were present in the MetS and Non‐MetS groups, accounting for 53.44% (70/131) and 49.56% (170/343); 41.22% (54/131) and 42.27% (145/343); and 5.34% (7/131) and 8.16% (28/343), respectively. The genotypes CC, CT, and TT of rs700518 were present in the MetS and Non‐MetS groups, accounting for 21.37% (28/131) and 20.12% (69/343); 60.31% (79/131) and 48.10% (165/343); and 18.32% (24/131) and 31.78% (109/343), respectively. All genotypes had statistically significant differences between the diseased and control groups (in all cases, *p *≤* *0.001). However, only the genotype TT of rs700518 was independently associated with MetS (OR = 2.181, 95% CI: 1.246–3.818, *p *=* *0.006) after adjusting for age.

**Table 6:** The genotypes and allele distributions of rs700518 and rs4646 in MetS and Non‐MetS groups and the comparison between the two groups

SNP	Type	MetS, ratio (%)	Non‐MetS, ratio (%)	*p*‐value^a^	Model	OR (95% CI)	*p*‐value^b^
rs4646	CC	70/131 (53.44)	170/343 (49.56)	**<0.001**		0.981 (0.612–1.574)	0.937
	CA	54/131 (41.22)	145/343 (42.27)	**<0.001**		.	.
	AA	7/131 (5.34)	28/343 (8.16)	**0.001**	Allele	0.968 (0.375–2.499)	0.947
rs700518	CC	28/131 (21.37)	69/343 (20.12)	**<0.001**	Allele	1.190 (0.678–2.091)	0.544
	CT	79/131 (60.31)	165/343 (48.10)	**<0.001**		.	.
	TT	24/131 (18.32)	109/343 (31.78)	**<0.001**		2.181 (1.246–3.818)	**0.006**

MetS, metabolic syndrome; SNP, single nucleotide polymorphisms; OR, odds ratio; CI, confidence interval. The boldface represents statistical significance (*p *<* *0.05).

a Chi‐square test.

b Multivariate logistic regression analysis, adjusted for age.

### The genotypes and allele distributions of rs700518 and rs4646 in MetS‐BPH and Non‐MetS‐BPH group and the comparison between the two groups

As shown in Table 7, regarding the SNPs of CYP19A1 gene, the genotypes CC, CA, and AA of rs4646 were present in the MetS‐BPH and Non‐MetS‐BPH groups, accounting for 54.26% (51/94) and 49.74% (189/380); 38.30% (36/94) and 42.89% (163/380); and 7.45% (7/94) and 7.37% (28/380), respectively. The genotypes CC, CT, and TT of rs700518 were present in the MetS‐BPH and Non‐MetS‐BPH groups, accounting for 21.28% (20/94) and 20.26% (77/380); 59.57% (56/94) and 49.47% (188/380); and 19.15% (18/94) and 30% (115/380), respectively. All genotypes had statistically significant differences between the diseased and control groups (in all cases, *p *≤* *0.001). However, only the genotype TT of rs700518 was independently associated with MetS‐BPH (OR = 2.111, 95% CI: 1.107–4.026, *p *=* *0.023) after adjusting for age.

**Table 7:** The genotypes and allele distributions of rs700518 and rs4646 in MetS‐BPH and Non‐MetS‐BPH groups and the comparison between the two groups

SNP	Type	MetS‐BPH, ratio (%)	Non‐MetS‐BPH, ratio (%)	*p*‐value^a^	Model	OR (95% CI)	*p*‐value^b^
rs4646	CC	51/94 (54.26)	189/380 (49.74)	**<0.001**		0.58 (0.216–1.556)	0.613
	CA	36/94 (38.30)	163/380 (42.89)	**<0.001**		.	.
	AA	7/94 (7.45)	28/380 (7.37)	**0.001**	Allele	0.872 (0.512–1.483)	0.279
rs700518	CC	20/94 (21.28)	77/380 (20.26)	**<0.001**	Allele	1.214 (0.648–2.274)	0.546
	CT	56/94 (59.57)	188/380 (49.47)	**<0.001**		.	.
	TT	18/94 (19.15)	115/380 (30)	**<0.001**		2.111 (1.107–4.026)	**0.023**

BPH, benign prostatic hyperplasia; MetS, metabolic syndrome; MetS‐BPH, BPH combined with MetS; MetS‐BPH, MetS combined with BPH; SNP, single nucleotide polymorphisms; OR, odds ratio; CI, confidence interval. The boldface represents statistical significance (*p *<* *0.05).

a Chi‐square test.

b Multivariate logistic regression analysis, adjusted for age.

### Association between the diseases of BPH, MetS, and MetS‐BPH and the related parameters of sex hormone, BPH, and MetS in the genotype TT of rs700518

As shown in Table 8, this study further analyzes the relationship between the genotype TT of rs700518 and all of the related parameters of sex hormone, BPH, and MetS. Our findings are as follows: Only E2 had a common independent association with the genotype TT of rs700518 in the BPH group (OR = 2.408, 95% CI: 1.474–3.013, *p *=* *0.007), MetS group (OR = 1.208, 95% CI: 0.461–2.714, *p *<* *0.001), and MetS‐BPH group (OR = 1.186, 95% CI: 0.437–3.222, *p *<* *0.001). However, IPSS (OR = 1.221, 95% CI: 1.128–1.322, *p *<* *0.001) and PSA (OR = 0.701, 95% CI: 0.498–0.982, *p *=* *0.042) had independent associations with the genotype TT of rs700518 in the BPH group, and FPG (OR = 2.603, 95% CI: 1.106–6.123, *p *=* *0.028) and PSA (OR = 0.298, 95% CI: 0.1.4–0.856, *p *=* *0.024) had independent associations with the genotype TT of rs700518 in the MetS‐BPH group.

**Table 8:** Association between genotype TT of rs700518 and related parameters of sex hormone, BPH, and MetS

rs700518 type TT (*n* = 133)																		
Parameters	BPH						MetS						MetS‐BPH					
	*n*	Value	*p*‐value^c^	OR	*p*‐value^d^	95% CI	*n*	Value	*p*‐value^c^	OR	*p*‐value^d^	95% CI	*n*	Value	*p*‐value^c^	OR	*p*‐value^d^	95% CI
Age, years																		
(+)^a^	89	70.88 ± 8.13	0.069	1.076	**0.026**	1.009–1.148	24	68.46 ± 8.82	0.362	0.972	0.442	0.906–1.044	18	69.11 ± 7.52	0.622	0.997	0.938	0.920–1.080
(−)^b^	44	68.07 ± 8.35					109	70.28 ± 8.16					115	70.08 ± 8.41				
TT, ng/mL																		
(+)^a^	89	4.80 ± 1.58	0.668	1.011	0.953	0.699–1.464	24	4.64 ± 1.56	0.489	0.833	0.415	0.536–1.294	18	4.68 ± 1.61	0.639	0.856	0.562	0.506–1.447
(−)^b^	44	4.93 ± 1.60					109	4.89 ± 1.59					115	4.87 ± 1.58				
E2, pg/mL																		
(+)^a^	89	46.73 ± 16.38	**0.018**	2.408	**0.007**	1.474–3.013	24	46.80 ± 21.32	**0.035**	1.208	**<0.001**	0.461–2.714	18	47.12 ± 22.13	**0.043**	1.186	**<0.001**	0.437–3.222
(−)^b^	44	40.43 ± 15.16					109	39.27 ± 14.25					115	39.61 ± 14.60				
INS, μIU/mL																		
(+)^a^	89	49.05 ± 28.73	0.532	1.01	0.716	0.958–1.065	24	55.10 ± 29.51	0.401	0.996	0.897	0.942–1.054	18	50.25 ± 27.84	0.984	1.013	0.698	0.950–1.080
(−)^b^	44	53.07 ± 37.36					109	49.34 ± 32.26					115	50.40 ± 32.44				
FPG, mmol/L																		
(+)^a^	89	5.48 ± 1.62	0.884	1.308	0.482	0.619–2.767	24	6.60 ± 2.44	**0.011**	1.59	0.138	0.862–2.932	18	6.74 ± 2.59	**0.028**	2.603	**0.028**	1.106–6.123
(−)^b^	44	5.44 ± 1.16					109	5.21 ± 1.03					115	5.26 ± 1.11				
SHBG, nmol/L																		
(+)^a^	89	74.68 ± 33.45	0.673	0.991	0.243	0.975–1.006	24	70.76 ± 30.18	0.404	1	0.993	0.982–1.018	18	70.46 ± 28.12	0.425	0.992	0.478	0.970–1.014
(−)^b^	44	77.82 ± 43.12					109	76.81 ± 38.13					115	76.55 ± 38.00				
DHT, pg/mL																		
(+)^a^	89	396.18 ± 321.83	0.457	1	0.705	0.999–1.001	24	427.39 ± 215.90	0.666	1	0.987	0.999–1.001	18	421.64 ± 231.39	0.806	1	0.992	0.999–1.001
(−)^b^	44	433.00 ± 235.40					109	404.25 ± 311.02					115	406.37 ± 305.11				
IPSS																		
(+)^a^	89	13.09 ± 8.23	**<0.001**	1.221	**<0.001**	1.128–1.322	24	11.33 ± 8.40	0.444	1.012	0.685	0.954–1.074	18	12.83 ± 8.26	0.15	1.048	0.198	0.976–1.126
(−)^b^	44	4.14 ± 5.32					109	9.86 ± 8.54					115	9.70 ± 8.49				
Qmax, mL/sec																		
(+)^a^	89	17.39 ± 5.70	0.378	1.134	**0.01**	1.030–1.249	24	14.83 ± 5.48	**0.032**	0.9	0.067	0.804–1.007	18	15.12 ± 5.10	0.095	0.963	0.545	0.852–1.088
(−)^b^	44	16.49 ± 5.35					109	17.59 ± 5.50					115	17.40 ± 5.61				
PSA, ng/mL																		
(+)^a^	89	1.33 ± 1.34	**0.042**	0.701	**0.042**	0.498–0.987	24	1.27 ± 1.44	0.535	0.872	0.558	0.552–1.379	18	0.91 ± 0.61	**0.003**	0.298	**0.024**	0.104–0.856
(−)^b^	44	1.68 ± 1.67					109	1.49 ± 1.47					115	1.53 ± 1.54				
TPV, mL																		
(+)^a^	89	22.51 ± 6.80	**0.031**	1.056	0.156	0.980–1.137	24	21.81 ± 5.91	0.934	1.002	0.967	0.917–1.094	18	21.25 ± 6.25	0.737	0.985	0.79	0.881–1.101
(−)^b^	44	20.12 ± 5.43					109	21.70 ± 6.60					115	21.79 ± 6.52				
HOMA‐IR																		
(+)^a^	89	1.75 ± 1.35	0.533	0.587	0.44	0.152–2.270	24	2.46 ± 2.18	**0.014**	1.084	0.912	0.259–4.548	18	2.29 ± 2.21	0.305	0.491	0.376	0.101–2.377
(−)^b^	44	1.93 ± 1.64					109	1.66 ± 1.20					115	1.73 ± 1.29				

BPH, benign prostatic hyperplasia; MetS, metabolic syndrome; MetS‐BPH, BPH combined with MetS; TT, total testosterone; E2, estradiol; DHT, dihydrotestosterone; INS, insulin; FPG, fasting plasma glucose; SHBG, androgen‐binding globulin; IPSS, international prostate symptom score; Qmax, maximum urinary flow rate; PSA, prostate‐specific antigen; TPV, total prostate volume; HOMA‐IR, insulin resistance index; SNP, single nucleotide polymorphisms; OR, odds ratio; CI, confidence interval. The boldface represents statistical significance (*p* < 0.05).

a Stands for BPH, MetS, or MetS‐BPH.

b Stands for Non‐BPH, Non‐MetS, or Non‐MetS‐BPH.

c Student's *t*‐test.

d Multivariate logistic regression analysis.

## Discussions

In this study, we evaluated the relationship between the polymorphisms of CYP19A1 gene in middle‐aged and elderly men and multiple pleomorphic hormones, including estrogen, in patients with BPH, MetS, and MetS‐BPH. We found that the value of E2 was higher in the diseased groups (including BPH, MetS, and MetS‐BPH groups) compared with the corresponding control groups (including Non‐BPH, Non‐Mets, and Non‐Mets‐BPH groups). The difference was statistically significant. Also, E2 had an independent association with BPH, MetS, and MetS‐BPH. Meanwhile, the increase in IR incidence is similar to that of E2. However, only MetS‐BPH had independent contact with IR. Regarding SNPs of CYP19A1 gene, both the genotypes CC, CA, and AA of rs4646 and the genotypes CC, CT, and TT of rs700518 were present in every group, and all genotypic distribution had statistically significant differences between the diseased and corresponding control groups. However, only the genotype TT of rs700518 was independently associated with BPH, MetS, and MetS‐BPH after adjusting for age. In the population of genotype TT of rs700518, we further analyzed the relationship between the parameters and found that the results of E2 were similar to those of the above. So, we are of the view that an SNP of CYP19A1 gene was the independent risk factor of MetS‐BPH, and we speculate that the pathophysiological mechanism of MetS‐BPH was achieved by this gene's regulation of estrogen metabolism.

Growing evidences from epidemiology and histopathology have confirmed that MetS is associated with BPH (Haider *et al*., 2009; Abdollah *et al*., 2011; Corona *et al*., 2014; Cyrus *et al*., 2014; Pan *et al*., 2014; Gacci *et al*., 2015). We had investigated whether MetS was related to lower urinary tract symptoms (LUTS) resulting from BPH (Zhao *et al*., 2016). In this study, we went further to detect the middle‐aged male crowd SNPs (rs4646 and rs700518) of CYP19A1 gene, multiple sex hormone indexes as well as BPH‐ and MetS‐related parameters. Also, we attempt to further evaluate the distribution of CYP19A1 SNPs in middle‐aged and older men and to understand how it affects MetS‐BPH by regulating some hormones (especially estrogen) metabolism. According to our examination results, these measurements were statistically significant for the increases in E2, FPG, IPSS, TPV, and HOMA‐IR in BPH patients compared with Non‐BPH patients. However, TT, INS, SHBG, DHT, Qmax, and PSA had no statistically significant differences. Also, E2, IPSS, TPV, and Qmax had independent associations with BPH (see Table 2). Further, the measurements show an increase in E2, INS, FPG, IPSS, and HOMA‐IR as well as a reduction in TT, SHBG, Qmax, and PSA in MetS patients compared with Non‐MetS patients. The differences were statistically significant. However, DHT and TPV had no statistically significant differences. E2 had an independent association with MetS (see Table 3). In addition, the measurements show an increase in E2, INS, FPG, IPSS, TPV, and HOMA‐IR as well as a reduction in TT, SHBG, Qmax, and PSA in MetS‐BPH patients compared with Non‐Mets‐BPH patients. The differences were statistically significant. However, the difference in DHT was not statistically significant. Also, TT, E2, IPSS, Qmax, PSA, and HOMA‐IR were independently associated with MetS‐BPH (see Table 4). So, we can conclude that E2 is a common independent associated risk factor of BPH, MetS, and MetS‐BPH. Moreover, we speculate that the changes in sex hormone metabolism and the rise in estrogen may play a key role in the pathogenesis of BPH, MetS, and MetS‐BPH in middle‐aged and elderly men. Also, IR may play a key role in promoting the progress of BPH and MetS. In most previous studies, estrogen and IR were isolated to compare BPH and MetS (Calais Da Silva *et al*., 1997; Simpson *et al*., 2002; Haider *et al*., 2009; Parsons, 2010; Abdollah *et al*., 2011; Corona *et al*., 2014; Pan *et al*., 2014; Dogan *et al*., 2015; Gacci *et al*., 2015; Ryl *et al*., 2015; Speakman *et al*., 2015; Telli *et al*., 2015); however, we did a systematic study of the relationship between MetS and BPH.

In terms of SNP, besides CYP19A1 gene, we also analyzed the androgen receptor (AR) gene, CYP11A1 gene, CYP17A1 gene, CYP1B1 gene, ESR1 gene, IGF‐1R gene, INSL3 gene, KLK3 gene, SHBG gene, SIRT1 gene, and SRD5A2 gene (data not shown in this paper). However, only the CYP19A1 gene was found to be valuable. At the same time, our research shows that the genotypes CC, CA, and AA of rs4646 and the genotypes CC and CT of rs700518 were not associated with BPH, MetS, and MetS‐BPH, and only the genotype TT of rs700518 was independently associated with BPH, MetS, and MetS‐BPH after adjusting for age (see Tables 5, 6, 7). In other words, the population distribution of genotype TT of rs700518 is a common independent associated risk factor of BPH, MetS, and MetS‐BPH. Our study further analyzed the relationship between all of the related parameters of sex hormone and BPH, MetS, and MetS‐BPH in the population distribution of genotype TT of rs700518, and the findings show that although IPSS and PSA were independently associated with BPH patients, and FPG and PSA were independently associated with MetS‐BPH patients, E2 had a common independent association with all BPH, MetS, and MetS‐BPH patients. Moreover, in the genotype TT of rs700518 population, E2 was significantly higher in BPH (46.73 ± 16.38 pg/mL), MetS (46.80 ± 21.32 pg/mL), and MetS‐BPH (47.12 ± 22.13 pg/mL) groups than the overall average of 38.62 ± 17.43 pg/mL (see Tables 8 and 1). Compared with previous research (Chen *et al*., 1988; McTernan *et al*., 2000; Simpson *et al*., 2002; Burnett‐Bowie *et al*., 2009; Santen *et al*., 2009; Balistreri *et al*., 2011; Berges *et al*., 2011; Ho & Habib, 2011; Cornu *et al*., 2017), our results illustrate the CYP19A1 gene single nucleotide polymorphisms in the Chinese population distribution and reveals that the genotype TT of rs700518 may be a common susceptible factor of BPH, MetS, and MetS‐BPH. Also, it is done by increasing the metabolism of estrogen.

**Figure 1:**
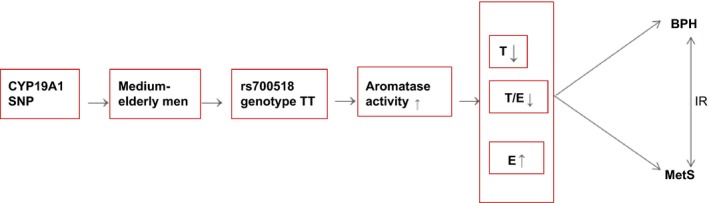
The schematic diagram for pathophysiological mechanism of benign prostatic hyperplasia combined with metabolic syndrome. SNP, single nucleotide polymorphism; BPH, benign prostatic hyperplasia; T, testosterone; E, estrogen; IR, insulin resistance; MetS, metabolic syndrome. [Colour figure can be viewed at wileyonlinelibrary.com]

According to the above analysis, it is not difficult to find that the increase in E2 was associated with all three diseases of BPH, MetS, and MetS‐BPH based on the metabolic indicators. There is a significant correlation between IR and MetS‐BPH. At the same time, in terms of genetics, we found that the gene polymorphism genotype TT of rs700518 of CYP19A1 was associated with the three diseases of BPH, MetS, and MetS‐BPH. We had to further analyze the relationship between the metabolic indicators and the three diseases in the rs700518 genotype TT populations, and the same result was obtained, which is that the rise in E2 has obvious correlation with the three diseases. Therefore, we conclude that the genotype TT polymorphism in rs700518 is a common risk factor of BPH, MetS, and MetS‐BPH. Moreover, it is achieved through the metabolism of E2. Meanwhile, in this process, IR plays an important role in MetS‐BPH. So, its possible mechanism is shown in Fig. 1, which is through CYP19A1 SNPs, especially through increasing the genotype TT of the rs700518 gene expression, which causes the metabolism level of estrogen to increase, leading to a reduction in the T/E ratio; this triggers IR, raises blood glucose levels, and promotes hyperplasia of prostate gland acinar cells and stromal cells in the prostate tissue, leading to the occurrence of clinical BPH and MetS. In contrast, BPH can lead to a systemic metabolic disorder, which can lead to IR, resulting in MetS (Crawford *et al*., 2006; Balistreri *et al*., 2011; Ersekerci *et al*., 2015; Cornu *et al*., 2017). Moreover, through IR, MetS and BPH interact and progress clinically. Probably, small doses of aromatase inhibitor therapy may have preventive and therapeutic effects on BPH and MetS in clinical procedures (Burnett‐Bowie *et al*., 2009; Ho & Habib, 2011).

The present study has some limitations. This study only involved the Chinese, with a majority of Han Chinese and a small number of Hui, Miao, Uighur, and Tibetan. There are differences in gene polymorphism of different ethnic groups, and we did not take that into consideration in the study. Our research population is from a single‐center data, and the sample size is relatively small, especially in some groups, such as MetS and MetS‐BPH groups. These limitations may influence our judgment of the actual results and conclusions. More central and large case studies are needed.

## Conclusions

This study systematically analyzed single nucleotide polymorphisms of CYP19A1 gene in BPH, MetS, and MetS‐BPH populations and found that the genotype TT of rs700518 is an independent risk factor of MetS‐BPH. Also, E2 increased in the population of MetS‐BPH, and the incidence of IR increased in the MetS‐BPH population compared with the Non‐Mets‐BPH population. The change was statistically significant. Thus, we speculate that the pathophysiological mechanism of MetS‐BPH may be through CYP19A1 SNPs, especially through increased genotype TT of rs700518 gene expression, which promotes the conversion of estrogen, leading to decreased T/E ratio; this triggers IR, leading to the occurrence of MetS‐BPH. Moreover, through IR, MetS and BPH interact and progress together in clinical procedures.

## Funding

This study was supported by funding from Beijing Municipal Science & Technology Commission (NO. Z141100002114005) as well as grants from National Natural Science Foundation of China (No. 71432002) and China Railway Corporation (No. 2014Z005‐C).

## Conflict of Interest

The authors declare that they have no conflict of interest.
